# Apnea and Diffuse Alveolar Hemorrhage Caused by Cocaine and Heroin Use: A Case Report

**DOI:** 10.5811/cpcem.2020.7.48438

**Published:** 2020-10-05

**Authors:** Gideon Logan, Ernesto Robalino, Tracy MacIntosh, Latha Ganti

**Affiliations:** * University of Central Florida College of Medicine, Department of Emergency Medicine, Orlando, Florida; † University of Central Florida College of Medicine, Department of Internal Medicine, Orlando, Florida

**Keywords:** opioid use, cocaine use, overdose, toxidrome

## Abstract

**Introduction:**

Drug overdose represents a growing reason for emergency department visits and hospitalizations in the United States. Co-ingestion of multiple substances is also on the rise, and toxidromes can be seen from any of multiple drugs in a single patient.

**Case Report:**

We present a case of diffuse alveolar hemorrhage secondary to cocaine abuse in a patient who was apneic and unresponsive after heroin overdose. The patient responded to supportive care and was discharged with complete return to physical and mental baseline.

**Conclusion:**

Clinicians must be vigilant for any number of concomitant toxidromes when a patient is brought in with complications following drug overdose.

## INTRODUCTION

Heroin is a semisynthetic drug able to cross the blood-brain barrier with affinity for *mu* opioid receptors producing analgesia and euphoria. Cocaine is a plant-derived stimulant drug that blocks the re-uptake of serotonin, dopamine, and norepinephrine, among other modes of action. Co-ingestion of heroin with cocaine remains a growing sector of illicit drug use and represents a poor prognostic indicator for drug addiction recovery from either substance individually, with or without medication-assisted treatment.^1^

While the euphoric effects of heroin and cocaine co-ingested have been described as synergistic, the toxic effects remain additive depending on the total dose, route, form, and frequency of use.^1^ Opioid overdose causes miosis and respiratory depression leading to hypoxemia, eventual coma, and then death. Cocaine abuse affects multiple organ systems including pulmonary effects, notably pulmonary hypertension, acute eosinophilic pneumonia, and diffuse alveolar hemorrhage (DAH).^2^ Here we present a case of heroin/cocaine co-ingestion causing initial apnea and hypoxemia, and then DAH requiring mechanical ventilation and supportive care.

## CASE REPORT

A 20-year-old male with a history of polysubstance abuse presented to the emergency department (ED) after being found unconscious by family members in a car. The patient was with his father and a cousin, and all three individuals were snorting both heroin and cocaine. Emergency medical services (EMS) reported the patient was apneic and had peripheral capillary oxygen saturation (SpO_2_) 65% at the time of arrival, but they were able to raise oxygen saturations to 92% with use of bag-valve-mask. EMS administered two doses of two milligrams intramuscular naloxone with spontaneous return of breathing and consciousness.

**Image 1:**
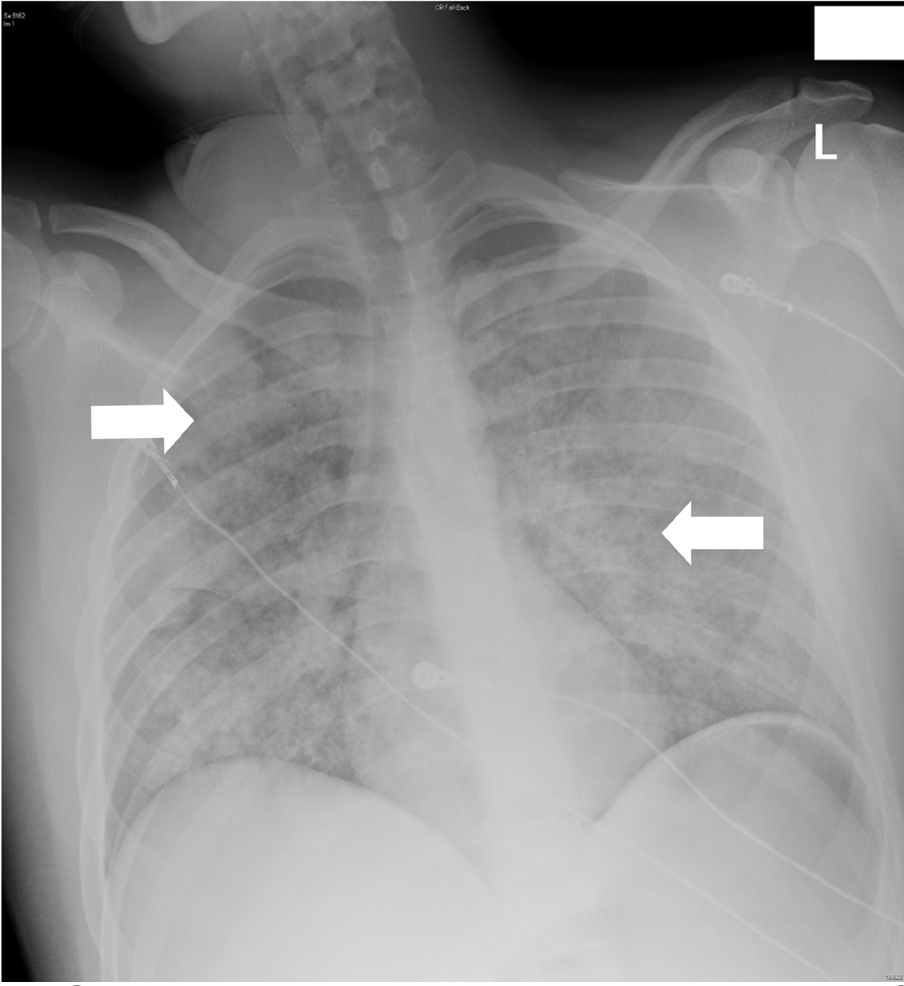
Chest radiograph at initial presentation showing diffuse pulmonary infiltrates. White arrows showing infiltrates.

On initial presentation to the ED, the patient’s vitals were as follows: heart rate 123 beats per minute; blood pressure 98/79 millimeters of mercury (mm Hg); respiratory rate 21 breathes per minute; SpO_2_ 100% on six liters per minute oxygen via nasal canula. and temperature 36.7° Celsius. The patient was awake and alert and able to answer questions with a primary complaint of chest tightness and persistent scant hemoptysis. The patient’s family noted epistaxis about five minutes after initial drug ingestion. After two liters of normal saline were bolused, the patient showed a reduction of pulse to 94 beats per minute and an increase in systolic blood pressure to116 mm Hg. On physical exam, no obvious sources of bleeding were identified intranasally, within the oropharynx or mouth. Auscultation of the lungs demonstrated coarse breath sounds in all lung fields. Initial blood laboratory tests, including a complete blood count and coagulation panel, were normal. Initial chest radiography demonstrated bilateral extensive alveolar densities (Image 1) The patient’s respiratory and mental status were maintained without need for additional doses of naloxone.

Three hours after arrival to the ED, the patient’s oxygen saturation began to decline, and he became tachypneic, tachycardic, and hypotensive. The patient required invasive mechanical ventilation for hypoxic acute respiratory failure. After initial intubation with confirmation of tube placement via capnography and radiography, achieving adequate oxygen saturation of >92% required acute respiratory distress syndrome protocol, volume-control ventilator settings: rate 24, tidal volume 440 milliliters, fraction of inspired oxygen (FiO_2_) 100%, positive end-expiratory pressure (PEEP) 15 centimeters water. The patient was then admitted to the medical-surgical intensive care unit for further management.

**Image 2:**
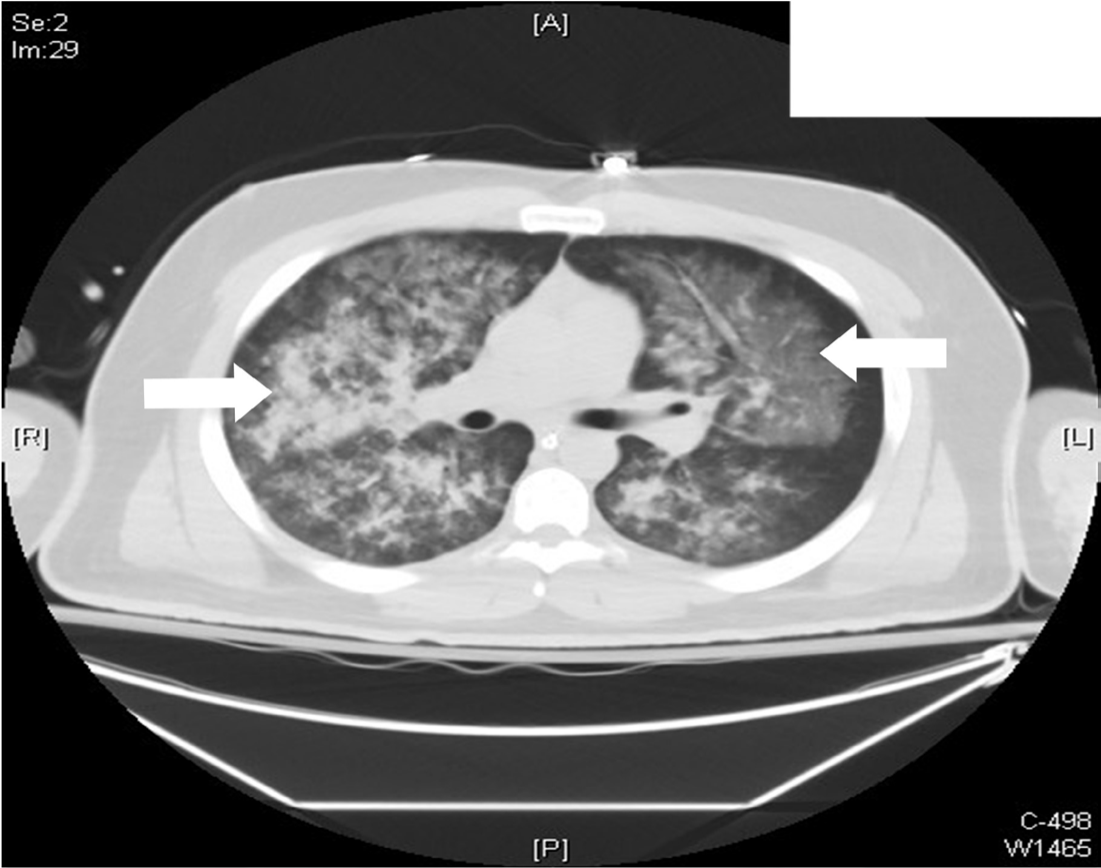
Non-contrast enhanced computed tomography of the chest showing diffuse infiltrates located centrally. White arrows showing infiltrates.

During his hospital stay, the patient remained hemodynamically stable, afebrile, without leukocytosis or lactic acidosis. Electrocardiogram (ECG) did not show evidence of ischemic changes and serial troponins were normal. Non-contrast enhanced computed tomography of the chest showed extensive bilateral diffuse pulmonary infiltrates with sparing of the periphery (Image 2).

CPC-EM Capsule

What do we already know about this clinical entity?

*Drug toxidromes are sometimes thought of as singular, but patients using multiple drugs can show multiple toxidromes at the same time*.

What makes this presentation of disease reportable?

*We present a case of apnea and altered mentation from heroin overdose that was reversed, followed by onset of diffuse alveolar hemorrhage from delayed cocaine toxicity*.

What is the major learning point?

Clinicians must keep suspicion for multiple toxidromes when symptoms are consistent with drug use or the patient is triaged as “overdose.”

How might this improve emergency medicine practice?

*“Red flag” history and physical exam findings should prompt attention for multiple toxidromes and adequate observation in drug use patients*.

Bronchoscopy revealed moderate to severe diffuse bronchial hyperemia and mucosal erosions, and successive aliquots of bronchoalveolar lavage showed progressively increasing blood-tinged secretions. Rheumatologic workup was unremarkable. Sputum and blood cultures were negative, and bronchial lavage cultures had no growth. The patient was treated empirically with piperacillin-tazobactam and azithromycin. After the first 24 hours with mechanical ventilation, the patient tolerated lower settings of FiO_2_ and PEEP and was later transitioned to non-invasive ventilatory support, with return to baseline ventilatory status and discharged home on hospital day six.

## DISCUSSION

In 2016, opioid overdose accounted for 200,322 ED visits and 85,944 hospitalizations, while cocaine overdose accounted for 19,709 ED visits and 8723 hospitalizations.^3^ Drug overdose will continue to affect every aspect of the healthcare system in the emergency, inpatient, and outpatient settings.

Alveolar hemorrhage is a respiratory emergency that needs to be recognized and treated early due to risks of high mortality.^4^ Clinical presentation can have a wide variety of symptoms including subtle symptoms of fever, dyspnea, and hemoptysis. In this case, multiple other etiologies were ruled out as causative of DAH,^5^ leaving cocaine-induced DAH the likely culprit. The ECG did not demonstrate ischemia, and serial troponins were negative ruling out cardiogenic sources of alveolar hemorrhage. Aspiration pneumonitis was considered given the altered and apneic initial presentation but was unlikely to have caused the extent of diffuse alveolar involvement seen. Rheumatologic markers were all negative making vasculitic etiologies unlikely, and cultures of sputum, blood, and bronchoalveolar lavage were negative for causative pathogens making infectious etiologies unlikely, although broad-spectrum antibiotics were started at first.

As demonstrated in the above case, co-ingestion of illicit drugs can be challenging to treat with multiple toxidromes playing out simultaneously in a single patient. What first presented as solely a heroin overdose with adequate reversal and stability quickly deteriorated secondary to cocaine-induced toxicity. While the treatment of opioid overdose is more straightforward with specific, targeted reversal agents and supportive care, the treatment of cocaine overdose lacks a targeted reversal agent and relies on the mainstay of supportive medications and treatments based on symptomatology. The exact emergency management for many drug overdose patients depends on practitioner preference and practice location, but a few key red-flag qualities that supported a more prolonged observation period in this case included knowledge of co-ingestion, degree of apnea and hypoxemia on initial presentation, and amount of naloxone required for reversal of respiratory depression and altered mental status. The presence of atypical physical exam findings (hemoptysis in this case) can also be a clue to severity and help guide decisions on disposition of overdose patients.

## CONCLUSION

Drug overdose is a common presenting complaint in many EDs and providers must address the toxicity from myriad abused substances, both illicit and prescribed. This case presented “routinely” with apnea and hypoxemia from heroin overdose that was corrected with naloxone but was complicated by diffuse alveolar hemorrhage caused by cocaine toxicity. While this case had a collateral historian to corroborate the details of the extent of the drug use, not all patients’ drug use will be so easily identified. Attention to any red-flag components of the history and unexpected physical exam findings will guide required interventions and disposition. Clinicians must maintain high suspicion for multiple toxidromes of co-ingested drugs when any drug overdose patient is treated.
